# Mothers and their children’s health (MATCH): a study protocol for a population‐based longitudinal cohort

**DOI:** 10.1186/s12884-021-03732-6

**Published:** 2021-04-12

**Authors:** Reihaneh Pirjani, Ashraf Moini, Javad Heshmati, Azar Mardi-Mamaghani, Mahnaz Esmaeili, Mehrnoosh Shafaatdoost, Arezoo Maleki-Hajiagha, Elham Karimi, Mojgan Hossein-boroujerdi, Fatemeh Shokri, Zahra Mosanezhad, Niloofar Bajool, Matina Noori, Ladan Hosseini, Emma Persad, Mahdi Sepidarkish

**Affiliations:** 1 Obstetrics and Gynecology Department, Tehran University of Medical Sciences, Tehran, Iran; 2 Breast Disease Research Center (BDRC), Tehran University of Medical Science, Tehran, Iran; 3 Department of Endocrinology and Female Infertility, Reproductive Biomedicine Research Center, Royan Institute for Reproductive Biomedicine, ACECR, Tehran, Iran; 4 Department of Nutritional Science, School of Nutritional Science and Food Technology, Kermanshah University of Medical Sciences, Kermanshah, Iran; 5 Department of Andrology, Reproductive Biomedicine Research Center, Royan Institute for Reproductive Biomedicine, ACECR, Tehran, Iran; 6 Department of Genetics, Reproductive Biomedicine Research Center, Royan Institute for Reproductive Biomedicine, ACECR, Tehran, Iran; 7 Department of Clinical Nutrition, School of Nutritional Sciences and Dietetic, Tehran University of Medical Sciences, Tehran, Iran; 8 Research Development Center, Arash Women’s Hospital, Tehran University of Medical Sciences, Tehran, Iran; 9 Department of Clinical Nutrition, School of Nutrition and Food Science, Food Security Research Center, Isfahan University of Medical Sciences, Isfahan, Iran; 10 Department of Health Education and Promotion, Iran University of Medical Sciences, Tehran, Iran; 11 Department Cell and Molecular Biology, College of BioScience, Islamic Azad University North Tehran Branch, Tehran, Iran; 12 Department for Evidence-based Medicine and Evaluation, Danube University Krems, Krems, Austria; 13 Department of Biostatistics and Epidemiology, School of Public Health, Babol University of Medical Sciences, Babol, Iran

**Keywords:** Life style, Maternal health, Maternal Nutritional physiological phenomena, Nutrition, Pregnancy, Pregnancy Outcome, Prenatal care

## Abstract

**Background:**

The quality of prenatal care is critical for the prevention of adverse pregnancy outcomes. However, according to the World Health Organization (WHO), only 64 % of women worldwide have access to over four sessions of prenatal care throughout their pregnancy. Thus, studies that address factors affecting maternal and child health status before and after pregnancy are of immense importance. The primary aim of the mothers and their children’s health (MATCH) cohort study is to evaluate the effect of nutrition, sleep quality, and lifestyle on maternal and neonatal outcomes.

**Methods:**

A prospective cohort of > 2500 pregnant women in the first trimester (before 12 weeks’ gestation) will be recruited at Arash Women’s Hospital in Tehran, Iran between February 2020 and August 2021. All eligible pregnant women will be followed from their first trimester of pregnancy until delivery at four time points and assessed through a series of in-person visits with interviewer-administered questionnaires and telephone interviews. Detailed data will be collected on maternal demographics, lifestyle, medical history, reproductive history, obstetric history, dietary intake, sleep pattern, blood specimens, and anthropometric measurements, alongside paternal demographics, lifestyle, and family history. The outcomes will include antenatal, peripartum, and postnatal maternal complications and infant growth and neurodevelopment.

**Discussion:**

The results of the MATCH cohort study will support the development of contextual interventions that can enhance antenatal, peripartum, and postnatal status, neonatal outcomes, and longevity mother and child.

## Background

Pregnancy is a high-risk period for expectant mothers and their unborn children and weighs significantly on family and community health status, potentially affecting the quality of life for future generations [1]. Unfortunately, over 830 pregnant women die every day throughout the world due to preventable adverse pregnancy outcomes [2]. According to a 2015 WHO report, 303,000 women died in 2015 due to pregnancy-related complications, and an additional 2.7 million cases of neonatal death and 2.6 million cases of stillbirth are reported annually. The maternal mortality ratio (MMR) varies from 239 to 100,000 live births in developing countries to 12 per 100,000 live births in developed countries. Ninety-nine percent of maternal deaths have been reported to occur in developing countries, with higher incidences in rural areas and low-income communities [3].

According to the 2015 WHO report, the major causes of maternal mortality during pregnancy and childbirth are preventable complications, including severe bleeding (mainly postpartum hemorrhage), infection (usually postpartum infections), hypertensive disorders, delivery complications, and unsafe induced abortion. Other causes are attributed to pre-existing disorders that have worsened or initially appeared during pregnancy [3]. The quality of prenatal and postnatal care is critical for the prevention of these complications, however only 64 % of women worldwide have access to over four sessions of prenatal care [4]. Improvement of maternal health is one of the essential priorities of the WHO with a goal to decrease MMR to fewer than 70 deaths per 100,000 live births globally by 2030. To reach this goal, the WHO works to implement clinical evidence-based guidelines and programs, set global standards, and provide technical support to Member States [3]. Maternal health has also been accepted as one of the United Nations Millennium Development goals, furthering international support for decreasing maternal mortality [5].

Due to these benchmark maternal health goals, studies addressing factors affecting maternal and child health status before and after pregnancy are of utmost importance, [6] and extensive new studies on adverse perinatal outcomes are warranted as risk factors continue to evolve [7, 8]. Evidence-based preventive and therapeutic decision-making, particularly for pregnant women, has become increasingly important in recent years due to the irreversibility of adverse pregnancy outcomes [9, 10].

Limited access and inadequate intake of well-balanced and nutrient-rich nutrition may affect maternal and child health. Few studies have investigated the dietary intake of pregnant women in Iran. Previous research has reported inadequate maternal energy and macronutrient intake. Further, low levels of micronutrients, namely iron, calcium, zinc, magnesium and copper, reported in both mothers and neonates, which may be indicative of the health status of women and neonates worldwide [11, 12]. The primary aim of the MATCH cohort study is to evaluate the effect of nutrition, sleep quality, and lifestyle on maternal and neonatal outcomes. The purpose of the present article is to explain the study protocol and methods used in this study.

## Methods

### Design overview

The MATCH cohort study will prospectively follow a cohort of > 2500 pregnant women in Tehran, Iran. The study will be conducted at the Arash Women’s Hospital which is affiliated with Tehran University of Medical Sciences. The Arash Women’s Hospital is one of the largest obstetrics and gynecology hospitals in Iran with approximately 2000 births per year. Enrollment of all eligible pregnant women will be referred before 12 weeks of gestation through partnerships with prenatal care providers. All eligible pregnant women will be followed from their first trimester of pregnancy until delivery by a series of face-to-face visits with interviewer administered questionnaires, anthropometric measurements, biospecimen collections, and phone conversations at four time points.

This study has been approved by the Ethics Committee of Tehran University of Medical Sciences. The identity number is IR.TUMS.MEDICINE.REC.1398.576. Before the study begins, written informed consent will be obtained from participants. Due to the length of the study, consent will be reobtained every six months.

### Eligibility and enrollment

All pregnant women of Iranian nationality aged 18 to 45 in their first trimester will be included in the study. Patients will be enrolled from February 2020 through August 2021. Exclusion criteria are the following: metabolic or chronic diseases, those following a special diet, the use of certain food supplements (except for pregnancy supplements, such as iron or folate) and physical, mental or cognitive impairments which preclude participation or informed consent.

The measurements and data collection time points throughout the project are described in Table 1. There are four data collection time points. Ten trained interviewers will collect the data during the weekdays with the exception of holidays).

**Table 1:** description of data elements and timeline

Methodology data	Phase 1 (6–12 weeks)	Phase 2 (24–26 weeks)	Phase 3 (32–34 weeks)	Phase 4 (12–72 h after delivery)	Data collection Methods	Measurement tool
Maternal demographics	**×**				Standardized interview	Standardized questions
Maternal lifestyle	**×**				Standardized interview	Standardized questions
Maternal medical history	**×**				Standardized interview	Standardized questions
Maternal reproductive history	**×**				Standardized interview	Standardized questions
Maternal obstetric history	**×**				Standardized interview	Standardized questions
Maternal family history		**×**			Standardized interview	Standardized questions
Maternal dietary intake	**×**				Standardized interview	Validated questionnaire (FFQ)
Maternal physical activity		**×**			Standardized interview	Validated questionnaire (IPAQ)
Maternal sleep pattern			**×**		Standardized interview	Validated questionnaire (Pittsburgh Sleep Quality Index) (Epworth Insomnia Severity Index)
Paternal demographics	**×**				Standardized interview	Standardized questions
Paternal lifestyle	**×**				Standardized interview	Standardized questions
Paternal medical history	**×**				Standardized interview	Standardized questions
Paternal family history	**×**				Standardized interview	Standardized questions
Maternal anthropometry	**×**				Calibrated Instruments and standardized Protocols	Digital weight scale (seca 813) Digital height scale (seca 206) Aneroid sphygmomanometer (seca b10)
Maternal blood specimens	**×**				Calibrated Instruments and standardized Protocols	Single central laboratory Automated hematology analyzer (sysmex corporation, kobe, Japan)
Infant anthropometry				**×**	Calibrated Instruments and standardized Protocols	Baby weight scale (seca 376) Baby height scale (seca 376)
Postnatal maternal complications				**×**	Standardized telephone interview	Standardized questions
Peripartum maternal complications				**×**	Standardized telephone interview	Standardized questions
Antenatal maternal complications				**×**	Standardized telephone interview	Standardized questions
Infant health				**×**	Standardized telephone interview	Standardized questions

Baseline data will be collected before the 12th week of gestation and provide information on maternal demographics, lifestyle, medical, reproductive, obstetric, social history, maternal nutritional status, and sleep patterns for one year before the pregnancy. Further, paternal demographics, social history, lifestyle, and family history data will be collected. Additional data including blood samples and anthropometric measurements will only be collected at the first prenatal visit. Patients will be surveyed a second time at 24 weeks of gestation (± 14 days). The data collected at this second point will provide information about maternal physical activity and details of maternal family history. Patients will be surveyed a third time at the 32 weeks of gestation (± 14 days). The data collected at this third point will include information about sleeping disorders during the early stages of the pregnancy. The final data collection point will be completed between 12 and 72 h after delivery, and will include data on birth outcomes, including gestational age, birth weight, and size, delivery type, and any intrapartum or postpartum maternal and neonatal complications. The study interviewers will maintain regular contact with women via telephone, email or text message and be notified if the participants give birth at another hospital.

### Quality assurance and quality control procedures

For quality assurance and quality control, we will employ proactive and thorough participant-tracking techniques. During the first visit, contact information, including home and work addresses, telephone numbers (home and work), cell phone number for both the participant and her husband, e-mail address and contact information for next of kin and a close friend will be obtained. During data collection, the quality of the data will be checked weekly to identify interviewer bias. 10 % of the data will be monitored and checked monthly by an independent researcher to ensure completeness. In order to ensure the reliability of the collected data, all interviewers will be trained prior to the start of the study using a variety of strategies, such as formal training sessions, practical demonstrations and role-plays, to learn how to explain the study objectives, take consent and administer the questionnaire. A training handbook will be used to ensure standardization of instructions and coherence of training norms for interviewers throughout the project.

### Socio‐economic, demographic and medical history variables

In the first visit, socio-economic and demographic data will be collected using a structured interview. Data will include date of birth, educational level, employment status, economic status based on home appliances [13], number of members in the household, homeownership status, and lifestyle variables, such as smoking, alcohol, and drug addiction. Past medical and surgical histories, alongside family history of diseases in first-degree relatives will be assessed. Details will also be recorded about reproductive history, including parity, gravidity, history of stillbirth or spontaneous abortion, previous modes of delivery, history of multiple births, family history of multiple births either in the participant’s family or her husband’s family, and history of adverse pregnancy outcomes, including pre-eclampsia, eclampsia, gestational diabetes, embryonic developmental disorder. Interviewers will also collect information related to infertility history, including duration, cause, and type of infertility, use of fertility treatments, and history of gamete or embryo donation.

### Nutrition: Food Frequency Questionnaire (FFQ)

Participants’ dietary intake will be evaluated during the first visit using the 168-item FFQ that contains questions about the type/brand of food, cooking methods, and frequency and amount of all foods and drinks they consumed during the one-year period leading up to the pregnancy. The FFQ was validated for the population in the Tehran Lipid and Glucose Study (TLGS), and its relative validity and reproducibility was proven [14]. For FFQ data, portion sizes will be converted to grams per week per food item by two experienced nutritionists.

### Maternal anthropometric measurements

Maternal height, waist circumference and hip circumference will only be measured at the first visit, but maternal weight will be measured at all four visits. Each measurement will be carried out twice and the mean will be recorded. If the second measurement is greater than or less than the 1.5 % standard deviation of the first measurement, a third measurement will be made and the midpoint measurement will be recorded. Weight will be measured with a digital weight scale (Seca 813) capable of weighing a maximum of 150 kg with an accuracy of 50 g. Before weighing, participants will be asked to remove their shoes, extra or heavy clothing, and empty their pockets. Infants will be weighed without clothes using a baby scale (Seca 376), with an accuracy of 10 g. Height measurements will be taken with shoes off in the standing position. Measurements will be taken twice during two separate visits using the height scale (Seca 206) with an accuracy of one millimeter. The average of the two measurements will be recorded. If the second measurement is greater than or less than the 1.5 % standard deviation of the first measurement, a third measurement will be made and the midpoint measurement will be recorded. The infant’s height will be measured by the infant’s recumbent length, using a horizontal length scale (Seca 376), with an accuracy of 1 mm. Blood pressure will be taken using a regularly tested aneroid sphygmomanometer (Seca b10) in a calm environment with suitable temperature. The participant will be seated in an upright position and asked to remove excess clothes that constrict arm blood flow. After relaxing for 3–5 min, blood pressure will be measured in the right arm, which will be placed on a table and raised to the level of the heart, with the back supported during the measurement. The measurement will be repeated about five minutes later, and the average of two measurements will be recorded. Participants will be asked to avoid intense activity, heavy meals, coffee, alcohol, stimulant drugs or drinks, and tobacco for at least half an hour prior to measuring blood pressure.

### Biospecimen collection procedures

Ten cubic centimeters of venous blood will be collected from the ulnar vein and mixed with an appropriate proportion of ethylenediaminetetraacetic acid (EDTA) anticoagulant. All blood samples will be analyzed in a single laboratory at Arash Hospital. Samples will be tested a maximum of four hours after sampling by an automated hematology analyzer (Sysmex Corporation, Kobe, Japan). Hematological indices in this study will include white blood cell count (WBC), red blood cell count (RBC), platelet count (Plt), hemoglobin (Hb), hematocrit (HCT), mean corpuscular volume (MCV), mean corpuscular hemoglobin (MCH), mean corpuscular hemoglobin concentration (MCHC), standard deviation and coefficient of variation of red cell distribution width (RCDW-SD and RCDW-CV), platelet distribution width (PDW), mean platelet volume (MPV), and platelet larger cell ratios (PLCR). At the same time, the differential count of white blood cells will be performed by peripheral blood smear stained with Giemsa color. The following laboratory parameters will be collected between 24 and 28 weeks of gestation: Hepatitis B virus (HBV) antigen (Ag), human immunodeficiency virus (HIV) antibody (Ab), hepatitis C virus (HCV) Ab, blood group, RH, indirect coombs, triglycerides (TG), total cholesterol, low-density lipoprotein (LDL) cholesterol, high-density lipoprotein (HDL) cholesterol, Hb A1C, fasting blood sugar (FBS), blood urea nitrogen (BUN), creatinine, urine culture, thyroid-stimulating hormone (TSH), 25 OH VitD3, and glucose tolerance test (GTT) 75 g or glucose challenge test (GCT) 50 g following by GTT 100 g in the case of abnormal GCT. For insurance of the accuracy of the results, the device will be calibrated by the reference methods prior to testing. Also, to ensure day-to-day consistency of the analytical process, a regular quality control program will be performed during the tests. We will determine the serum glucose level with the oxidase method using Hitachiauto analyzer model 7700 (Hitachi Medical, Tokyo, Japan). For GCT 50 g, a blood sample will be taken one hour after the consumption of a syrup containing 50 g of glucose. Participants who have a GCT between 140 and 200 mg/dl will undergo a 100 gram oral GTT. For GTT 75 g, after measurement of FBS, participants will be asked to drink a syrup containing 75 g of glucose. At one hour and two hours after consumption of the syrup, serum glucose levels will be measured again.

### Physical activity

The International Physical Activity Questionnaire (IPAQ) will be used to collect information on the quantity and quality of physical activity. Participants will record the amount of time spent on each activity level as the number of days in the last seven-day period and average amount of time per day. Physical activity quality will be listed as: vigorous (activities that require intense physical exertion and cause the participant to breathe much stronger than usual(, moderate (activities that take moderate physical effort and make the participant breathe somewhat harder than normal), walking (including at work and at home, walking to move from one location to another, and any other walk intended solely for recreation, sport, exercise or recreation), and sitting (including time spent at work, at home, while doing course work and during leisure time) [15, 16]. This physical activity information will be converted to Metabolic Equivalent of Tasks (METs). The METs or metabolic equivalent is a physiological measure expressing the energy cost of physical activity and is defined as the ratio of the metabolic rate in a given physical activity to the baseline metabolic rate. One MET shows the amount of energy consumed when sitting quietly at rest and is equivalent to 3.5 ml/kg/min of VO_2_ Max. [17].

### Sleep Quality

We will assess sleep quality during the third visit (32 weeks ± 12 days) via the Pittsburgh sleep quality index–19 items (PSQI-19), Epworth sleepiness scale (ESS), and insomnia severity index (ISI). The PSQI-19 is a widely used and well-validated19-itemself-rating questionnaire that measures clinical and subjective sleep complaints in adults during the previous month. PSQI-19 generates seven component scores, including subjective sleep quality, sleep latency, sleep duration, habitual sleep efficiency, sleep disturbances, use of sleeping medication, and daytime dysfunction. The PSQI-19 provides a global score ranging from no sleep difficulty to severe difficulties. A cut-off value of five has been shown to have a sensitivity of 89.6 % and a specificity of 86.5 % for the diagnosis of poor sleep quality [18, 19]. The ESS is a questionnaire that measures daytime sleepiness. The scores range from 0 to 24, and a total score of over 10 indicates excessive day time sleepiness [20, 21]. The ISI is a self-reportedseven-item questionnaire with scores ranging from 0 to 28 as follows: no insomnia (0–7), sub-threshold insomnia (8–14), moderate insomnia (15–21), and severe insomnia (22–28)[22, 23].

### Establishing the GA

Ultrasound scans for each participant will be performed through diagnostic medical sonography by an ultrasound technician five times during the pregnancy to monitor fetal development, possible abnormalities, and the exact GA. The initial ultrasound should occur between 6 and 12 weeks’ gestation. One sonographer will perform all scans to reduce the risk of measurement errors. The expected date of delivery (EDD) will be calculated according to Naegele’s rule, based on the first day of the last menstrual period (LMP) [24]. If a participant does not accurately recall the LMP or has irregular menstrual periods, the GA and EDD will be calculated based on the initial first trimester ultrasound. If the difference in GA between the patient’s LMP and initial ultrasound is more than five days in the first trimester, the GA and EDD will be calculated based on the initial ultrasound.

### Providing routine antenatal care for women

For all first time pregnant women with no complications, seven visits will be conducted. After the first visit at 6 to 10 weeks of pregnancy, subsequent visits will happen between weeks 16–20, 24–30, 31–34, 35–37, 38, 39 and 40. In the first visit, the following variables will be measured: menstrual status, gestational age and approximate date of delivery, history of underlying disease, previous history of genetic disease, history of high-risk behaviors in couples, history of psychiatric disorder and of intimate partner violence, drug use and drug sensitivity. Examinations performed during each visit will include monitoring vital signs, measuring weight, uterine height and determining gestational age, listening to the fetal heartbeat, recording fetal movements, and reviewing maternal complaints. During the first visit, the following tests will be performed: HBsAg, TSH, VDRL, HIV, CBC, BG, U/A, U/C, BUN, Cr, and FBS. An indirect Coombs assay may be used to determine if there are antibodies to factor Rh in the woman’s blood. Laboratory tests will be repeated at 24 to 28 weeks of pregnancy (preferably at 28 weeks) as follows: CBC, FBS,U/A and a second indirect Coombs assay. Targeted ultrasound will be performed at 16 to 18 weeks of gestation to assess placental migration and fetal abnormalities. Ultrasound will be performed at 31 to 34 weeks of gestation to assess the growth status of the fetus, placenta and amniotic fluid.

### Primary outcome measures

Adverse outcomes associated with pregnancy will be diagnosed and confirmed through examination by a perinatologist and laboratory assessments. The pregnancy outcomes considered in this study are as follows: spontaneous abortion, ectopic pregnancy, preterm pre-labor rupture of membranes (PPROM), type of delivery (vaginal, operative vaginal, or cesarean), gestational age at delivery, gestational hypertension, preeclampsia, HELLP (hemolysis, elevated liver enzyme levels, and low platelet levels) syndrome, preterm labor, gestational diabetes mellitus (GDM), anemia, acute fatty liver, polyhydramnios, oligohydramnios, placenta previa, placenta accreta, retained placenta, postpartum hemorrhage (PPH), postpartum infection and any other complication. Fetal measurements will include height, weight, and head circumference. Adverse fetal outcomes will include birth trauma, shoulder dystocia, intrauterine growth restriction (IUGR), intrauterine fetal death (IUFD), meconium aspiration syndrome, fetal distress syndrome, transient tachypnea of the newborn (TTN), apnea, neonatal intensive care unit (NICU) admission, macrosomia, low birth weight (LBW), prematurity, intraventricular hemorrhage (IVH) and any other complication.

### Power calculation and sample size

We calculated the sample size by considering a 5 % probability of detecting at least one adverse outcome in non-exposed pregnant women and with a prespecified RR = 2 as a measure of association. A total sample of 475 pregnant women per group including a 10 % dropout factor is assumed to provide 80 % power to detect a difference in the incidence of adverse event between the exposed and non-exposed groups; and a two-sided test having a type I error of 0.05.

### Statistical analysis

Data processing and statistical analysis will be performed using the Stata statistical package version 16 (Stata Corp LP, College Station, TX, USA). Analysis of baseline data will be conducted to describe the socio-demographics, nutritional status, and birth characteristics of pregnant women. The normality of data will be checked for extreme values and outliers using normality plots (histogram and Q-Q) and visual inspection. Data will be presented as mean scores ± SD for continuous data and as percentages for categorical data. Normally distributed data will be presented as means ± SD; non-normally distributed data will be expressed as medians (25th percentile, 75th percentile). Data will be analyzed by linear regression models, including analysis of variance (ANOVA), analysis of covariance (ANCOVA), multiple linear regression analysis and linear mixed-models. The level of significance will be set at p < 0.05.

## Discussion

The importance of perinatal status and its role in maternal and child health is indisputable. Strategies to promote nutritional interventions in community and facility settings have shown promising benefits for women and child health; however, evidence for the specific effects of micro-nutrients on pregnancy and neonatal outcomes is scarce [25]. The findings of a comprehensive review have highlighted ten evidence-based nutrition interventions to address nutrition and micronutrient deficiencies in women and children. The results emphasized that the current total number of deaths in children under five can be reduced by 15 % if pregnant women can access ten evidence-based nutritional interventions at 90 % coverage. The results further concluded that continued investments in nutrition-specific interventions can prevent maternal and child malnutrition and micronutrient deficiencies, alongside improving pregnancy outcomes. Thus, there is insufficient evidence about the interaction between various micro-nutrients and pregnancy outcomes [26]. A clear need exists to introduce promising evidence-based interventions during the preconception and prenatal period in countries highly burdened by poor nutrition and young maternal age of first pregnancies.

The authors of the aforementioned review proposed that future studies should focus on these aspects ensuring consistency in measurement and reporting of outcomes. With the MATCH study, we aim to evaluate the effect of nutrition, sleep quality, and lifestyle during antenatal and postpartum periods on mothers’ and newborns’ well-being. The most important strength of this study is the multifactorial approach to perinatal health combined with a longitudinal design with measurements during all trimesters of pregnancy. Another strength of the current study is the comprehensive assessment of risk factors related to adverse pregnancy outcomes. These factors include nutritional pattern, physical activity, sleep disturbances, medical history, previous pregnancy history, lifestyle, and socioeconomic status. In this study, data will be collected by trained interviewers, which considerably reduces and minimizes measurement error. Furthermore, due to of the large sample size, risk factors for rare pregnancy outcomes can be identified. The main limitation of the current study, like other cohort studies, is loss to follow up. However, through use of social networks, educational pamphlets, and continuous communication, we aim to minimize the attrition rate. Further, the site of this study is a specialized gynecological hospital, which may introduce selection bias which could reduce the generalizability of the study findings. However, as Arash Hospital is the largest maternity hospital in eastern Tehran, it treats all pregnant women from the province of Tehran and across the country of Iran, rendering the patient population diverse. In order to maintain consistency in data collection from a large patient population, this study was designed as a single-center study to preserve internal validity [27].

The MATCH prospective cohort study, which is representative of pregnant women in an Iranian urban setting, will expand our current knowledge about the casual effects of life style factors on prenatal and neonatal outcomes. The findings will promote evidence-based medicine, inform decision makers about the health and well-being of pregnant women and neonates, improve the counselling of future pregnant women and guide clinician management.

## Data Availability

Study data is available by corresponding author with rational request.

## Abbreviations

WHO: World health organization
MCHS: Maternal and child-health studies
MATCH: Mothers and their children’s health
MMR: Maternal mortality ratio
FFQ: Food Frequency Questionnaire
TLGS: Tehran Lipid and Glucose Study
WBC: White blood cells
RBC: Red blood cell
Plt: Platelet count
hemoglobin: Hemoglobin
HCT: Hematocrit
MCV: Mean corpuscular volume
MCH: Mean corpuscular hemoglobin
MCHC: Mean corpuscular hemoglobin concentration
RCDW: Red cell distribution width
PDW: Platelet distribution width
MPV: Mean platelet volume
OGTT: Oral glucose tolerance test
GCT: Glucose challenge test
IPAQ: International Physical Activity Questionnaire
Mets: Metabolic Equivalent of Tasks
PSQI-19: Pittsburgh sleep quality index–19 Items
ESS: Epworth sleepiness scale
ISI: Insomnia severity index
EDD: Expected date of delivery
LMP: Last menstrual period
EDTA: Ethylenediaminetetraacetic acid
PPROM: Preterm pre-labor rupture of membranes
GDM: Gestational diabetes mellitus
IUGR: Intrauterine growth restriction
IUFD: Intrauterine fetal death
LBW: Low birth weight
IVH: Intraventricular hemorrhage
TTN: Transient tachypnea of the newborn
ANOVA: Analysis of variance
ANCOVA: Analysis of covariance
